# Data showing the effects of vibratory disc milling time on the microstructural characteristics of Coconut Shell Nanoparticles (CS-NPs)

**DOI:** 10.1016/j.dib.2018.12.067

**Published:** 2018-12-24

**Authors:** Omolayo M. Ikumapayi, Esther T. Akinlabi

**Affiliations:** Department of Mechanical Engineering Science, University of Johannesburg, Auckland Park Kingsway Campus, Johannesburg, 2006, South Africa

**Keywords:** Coconut shell, EDX, Milling time, Nanoparticle, SEM, XRF

## Abstract

Coconut Shell (CS) as agricultural lignocellulosic biomaterial and agro-waste is predominantly available in India, Malaysia, Nigeria, Thailand, Sri Lanka, and Indonesia. It has proven to have effective durability characteristic, good abstractive resistance, high toughness, and good adsorption properties, and is most suitable for long standing use in many applications such as reinforcement, source of energy, fillers as well as activated carbon and its performance, efficiency and effectiveness depend wholly on whether is in form of nano-, micro-, and macro- particles. In this data, effects of milling time on morphological characteristics was experimented using Scanning Electron Microscopy (SEM), Energy Dispersive X-ray (EDX), and X-Ray Fluorescence (XRF) analyses. The SEM images were taken at magnifications of 1.00kx, 2.00kx and 5.00kx which gives respective 50 µm, 20 µm and 10 µm in different milling time of 0, 20, 40 and 60 mins. Digital Vibratory Disc Milling Machine (VDMM) rated 380 V/50 Hz at 940 rpm was employed for the grinding and the morphology of the milled nanoparticles were characterised. It was revealed from the data collected that 0 min (i.e. 75 µm sieved) has the highest mean area value of 16.105 µm^2^ and area standard deviation of 200.738 µm^2^ with least value of a number of particle size distribution of 809 µm. In contrast, 60 mins milled has the lowest values for mean area and area standard deviation of 8.945 µm^2^ and 115.851 µm^2^ respectively with the highest number of particle size distribution of 2032 µm. It was observed that milling time increases the number of particle sizes distributions and reduces the area of particle size.

**Specifications table**

**Table:** 

Subject area	Mechanical Engineering & Material Science
More specific subject area	Nanoparticle of Biomaterials
Type of data	Micrographs, graphs and table
How data was acquired	Coconut shell was obtained from coconut fruits. It was then crushed, grinded and milled into nano-size with the help of vibratory disc milling machine. The milled and unmilled samples were then taken for testing and characterisation using SEM-EDX as well as XRF
Data format	Raw and Analysed
Experimental factors	The milling time was taken at 0, 20, 40 and 60 min using Vibratory Disc Milling Machine (VDMM). The SEM images were taken at magnifications of 1.00kx, 2.00kx and 5.00kx for 50 µm, 20 µm and 10 µm respectively.
Experimental features	Micrographs of the milled and unmilled samples, elemental composition of tested samples at different milling times using EDX and also chemical compositions of the sample.
Data source location	University of Johannesburg, Johannesburg, South Africa
Data accessibility	The availability of the data is within the peripheral of this article

**Value of the data**

• The data is very valuable in a manner that the users will be able to know the variation in elemental compositions at different milling time as well as unmilled coconut shell nanoparticle (CS-NPs) as revealed by EDX.
• The data obtained can be used in investigating surface processing as well as aggregate, filler and reinforcement in both polymer and metal matrix composite at different milling time.
• The data revealed the micrographs of the milled and unmilled coconut shell at different milling time, the users will be able to see the trend and patterns of the milled coconut shell which can also predict the absorption rate of CS-NPs at any applications.
• The data also presented various chemical compositions that are in Coconut Shell powder as revealed by the XRF.

## 1 Data

The data presented are the characterizations derived from coconut shell powder in Fig. 2 that was milled using digital vibratory disc milling machine (VDMM) in Fig. 3. Chemical composition of the coconut shell powder is presented in Table 1 as analysed by X-Ray Fluorescence (XRF). Microstructural data from Scanning Electron Microscopy (SEM) in Fig. 4 were taken at different magnifications of 1.00kx, 2.00kx and 5.00kx are presented in Fig. 5. Statistical data from the coconut shell nanoparticles such as particle sizes, mean area, standard deviation at different milling time as analysed by ImageJ are shown in Table 3. Details variable elemental compositions from the investigated coconut shell nanoparticles at different milling time of 0, 20, 40 and 60 min as analysed by Energy Dispersive X-ray (EDX) are presented in Fig. 6 and Table 4.

**Fig. 1:**
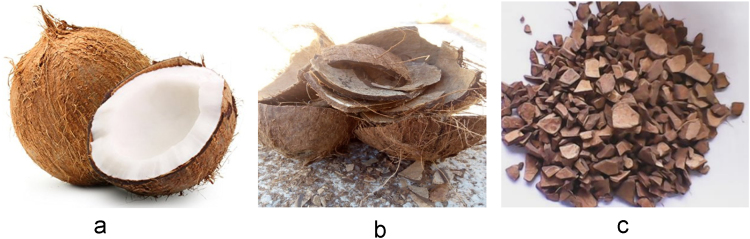
a: Coconut fruits b: Coconut Shell (CS) c: Broken CS.

**Fig. 2:**
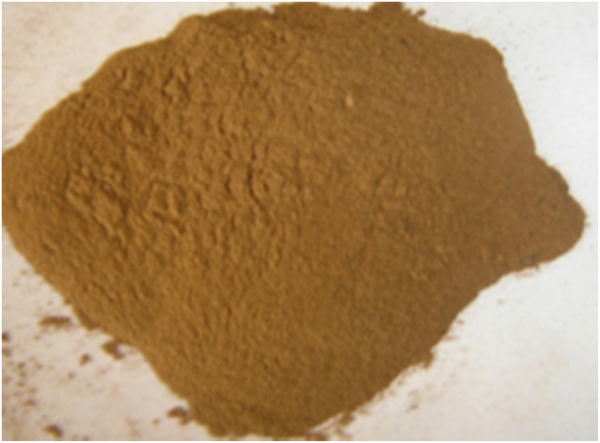
Grinded Coconut shell Powder (CS-P).

**Fig. 3:**
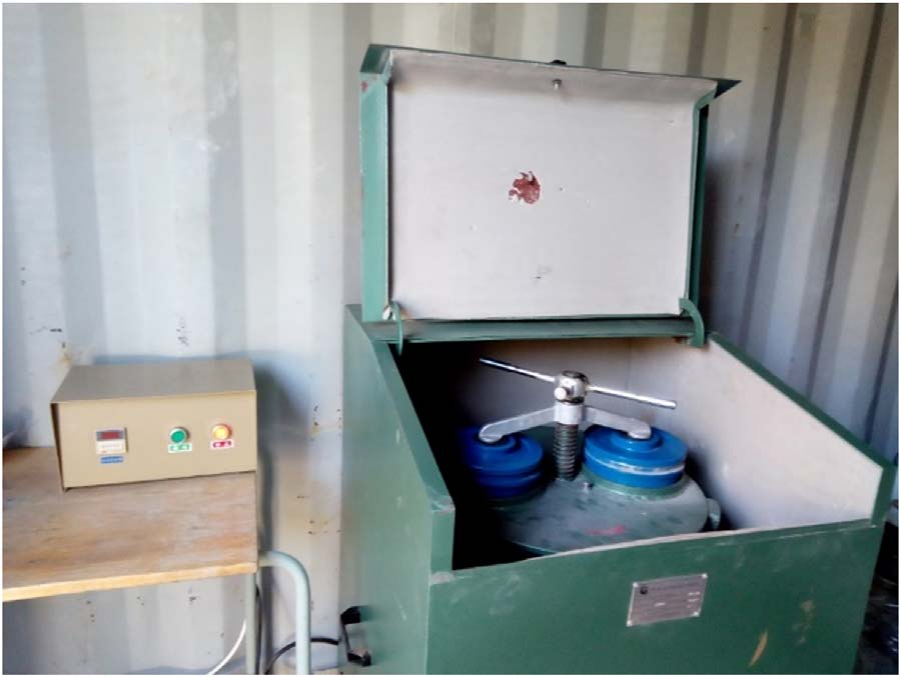
Diagram of vibratory disc milling machine.

**Table 1:** The chemical composition analysis of CS-NPs using XRF.

Chemical Formula	Al_2_O_3_	Ca0	MgO	K_2_O	Na_2_O	SiO_2_	Fe_2_O_3_	MnO	ZnO
CS-NPs (%)	16.76	0.78	19.4	0.42	0.41	45.6	8.98	0.17	0.39

**Fig. 4:**
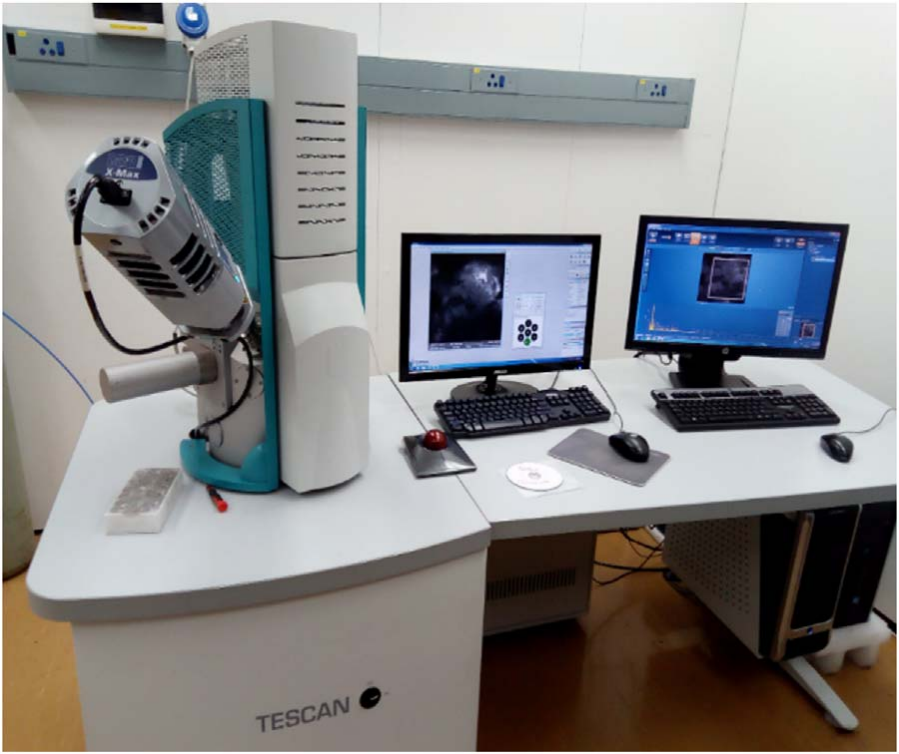
SEM-EDX analysis machine.

**Fig. 5:**
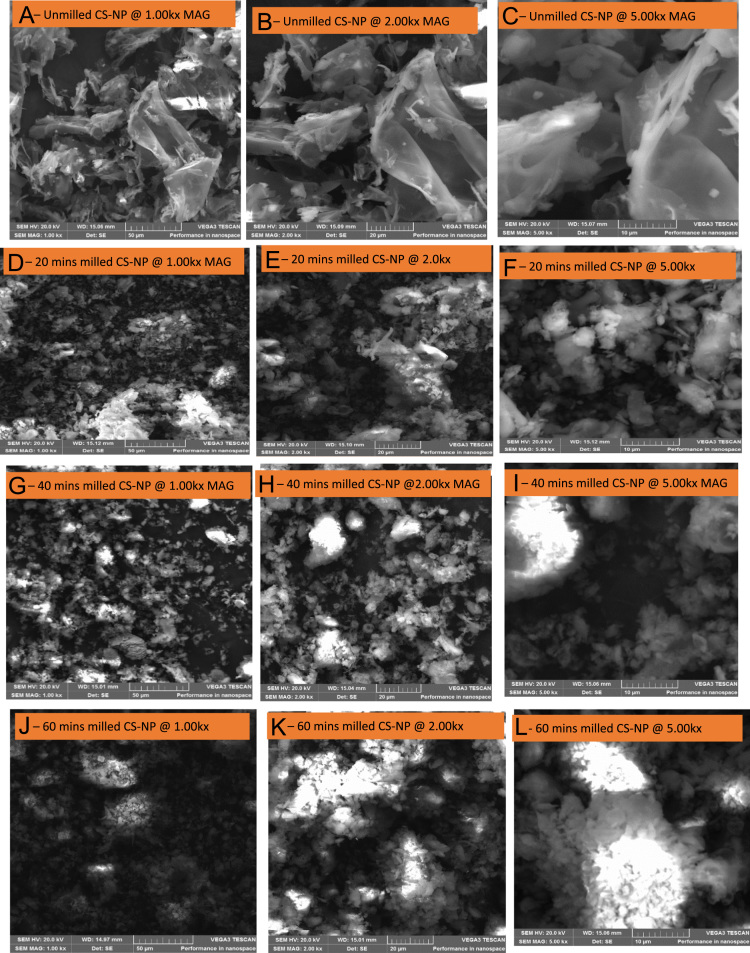
(A –C) Unmilled CN-NPs at 1.0kx, 2.0kx and 5.0kx respectively, (D – F) 20 min milled of CN-NPs at 1.0kx, 2.0kx and 5.0kx respectively, (G – I) 40 min milled of CN-NPs at 1.0kx, 2.0kx and 5.0kx respectively, (J – L) 60 min milled of CN-NPs at 1.0kx, 2.0kx and 5.0kx respectively.

## 2 Experimental design, materials and methods

Matured and edible Coconuts Fruits (Fig. 1a) were obtained from commercial market at Johannesburg, South Africa and the outer part of the coconut fruits (exocarp) were removed with the aid of cutlass after which they were then broken into smaller and irregular chunks in sizes which ranges from 2 to 10 mm (Fig. 1b), the coconut water inside of them were drained and the edible part of them (mesocarp) were removed from the hard and woody part (endocarp) which was known as coconut shell (CS) - lignocellulosic agro-waste [1], [2], [3], the shell was then washed and cleaned with deionized water and acetone to remove any unwanted contaminants (Fig. 1c) and this was then placed in an electric oven at 50 °C for 5 days to remove any traces of moisture that may be present before taken for crushing.

### 2.1 Experimental methodology

The coconut shell obtained were dried at 50 °C in an electric oven for 5 days to ensure effective and total dryness of any moisture that may likely present therein. The coconut shell were then taken for crushing with the aid of a crusher. The crusher was thoroughly washed, dried and cleaned with acetone before and after use to remove any contaminants within. The crusher was used to pulverized it into small macro particles, this was further milled into smaller particles and sieved using ASTM meshes standard range by employing KingTest Sieve of 75 µm size on KingTest Sieve Shaker (VB 200/300) having an operating voltage of 220 V/50 Hz and 5 A. The Coconut Shell powder (CS-P) (Fig. 2) proportion that passed through the mesh size of 75 µm was then taken for milling at different milling times.

### 2.2 X-ray fluorescence (XRF) analysis

In this research, PHILIPS PW1404 X-Ray Fluorescence (XRF) Spectrometer was used to analyse the chemical composition of CS-NPs after sieved with 75 µm ASTM standard sieve and the data gotten from the analysis is presented in Table 1.

### 2.3 Vibratory disc milling machine (VDMM)

Mechanical Dry milling (MDM) operation was performed using a digitalized vibratory disc milling machine (Model 2MZ-200). The machine was thoroughly washed, dried and cleaned with acetone before and after use to remove any contaminants that may be present. 30 g of coconut samples were charged into each bowl and then set for running. The machine was interrupted every 5 min of operation in order to avoid a rise in temperature and at the same time to limit adherence of the powder within the container walls, the cooling interval before the next running was 20 min. The machine has specification as presented in Table 2 and the milling effects on the particle size and mean area of the sample is presented in Table 3 as analysed by ImageJ software.

**Table 2:** Vibratory disc milling machine specification.

Property	Specification
Dimension	740 × 740 × 950 mm
Number of bowls	2
Capacity per bowl	200 g
Feed Size	< 15 mm
Motor	380 V/ 50 Hz, 1.5 KW
Motor speed	940 rpm

**Table 3:** Measurements of particle size area of CS-NPs at unmilled and varying milling time.

Milling Time (min)	Mean Area (µm^2^)	STD Area (µm^2^)	Min. Area (µm^2^)	Max. Area (µm^2^)	No. of Particle Sizes (µm)
0	16.105	200.738	0.074	4,988.486	809
20	14.384	178.957.	0.065	2,599.508	1379
40	12.528	147.684	0.057	908.293	1631
60	8.945	115.851	0.004	47.889	2032

It was revealed by ImageJ software processing analysis used for Table 3 data collection that at 0 min (i.e. 75 µm sieved) has the highest mean area value of 16.105 µm^2^ and area standard deviation of 200.738 µm^2^ with least value of a number of particle size distribution of 809 µm. In contrast, 60 mins milled has the lowest values for mean area and area standard deviation of 8.945 µm^2^ and 115.851 µm^2^ respectively with the highest number of particle size distribution of 2032 µm. It was observed that milling time increases the number of particle sizes distributions and reduces the area of particle size. It was revealed that progressive milling leads to an increase in particle size distributions and decrease in the area of the particle size as noted in Table 3.

### 2.4 Microstructural analysis

Coconut Shell Nanoparticles (CS-NPs) obtained were then characterised by the use of scanning electron microscopy (SEM) and Energy dispersive X-ray spectroscopy (EDXS). These tools were employed to determine the morphology and the elemental composition at different milling time of 20, 40, 60 min and unmilled samples. ImageJ software was employed to analyse the statistical measurement of the SEM Image as displayed in Table 3.

### 2.5 Data acquisition

In this data article, mechanical dry milling (MDM) was carried out with different process milling time of 20, 40 and 60 min and powder of CS-NPs were studied using SEM-EDS. It was established from the data collected that the durations of MDM affected volume, surface area, particle size, pore size distributions, microstructure and some other mechanical properties as well as morphology of the powders were affected.

### 2.6 Scanning electron microscope

**Fig. 6:**
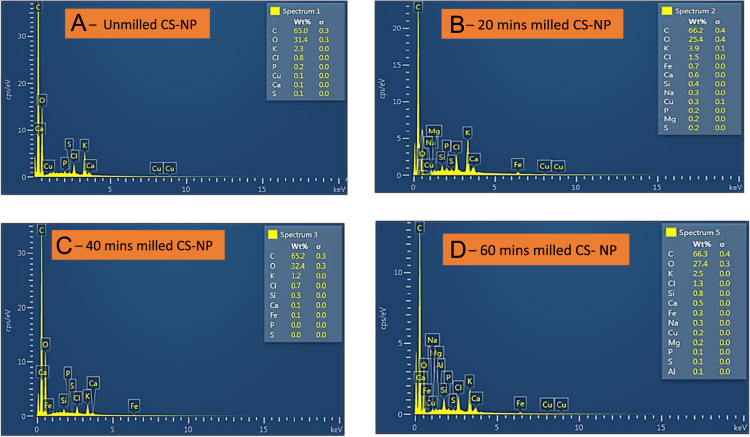
Elemental compositions at different milling time as analysed by EDXS.

TESCAN model, type VEGA 3 LMH and model no VG9731276ZA (Fig. 4) with the following details 50/60 Hz, 230 V and 1300 VA was the type of SEM machine that was employed for the studies. In order to have the sample more conductive and to have better resolution, the samples were sputter coated with a thin layer of carbon just before the scanning electron microscope analyses coupled with Energy Dispersive X-ray spectrometer (EDXS) analyses. The beam intensity used in the analysis was 12 and the accelerating voltage used was 20 kV, all micrographs were taken at SEM magnification of 1.00kx, 2.00kx and 5.00kx for different milling times of 20, 40 and 60 mins. The elemental compositions of CS-NPs was also analysed at different milling times was analysed by SEM. The SEM micrographs of different milling time were presented in Fig. 5 and the EDXS data were presented in Fig. 6 and the data collected has been presented in Table 4. Sizes of CS-NPs were determined using SEM/software.

**Table 4:** Variable in elemental compositions of CS-NPs as analysed by EDXS.

Powder	Element	Elemental composition at different milling times			
		0 min (Unmilled)	20 min	40 min	60 min
CS-NPs	C	65.0	66.2	65.2	66.3
	O	31.4	25.4	32.4	27.4
	K	2.3	3,9	1.2	2.5
	Cl	0.8	1.5	0.7	1.3
	Si	n. d	0.4	0.3	0.8
	Ca	0.1	0.6	0.1	0.5
	Fe	n. d	0.7	0.1	0.3
	Na	n. d	0.3	n. d	0.3
	Cu	0.1	0.3	n. d	0.2
	Mg	n. d	0.2	n. d	0.2
	P	0.2	0.2	n. d	0.1
	S	0.1	0.2	n. d	0.1
	Al	n. d	n. d	n. d	0.1

n. d ( not detected)

Fig. 5(A – C) represent the micrographs for the unmilled Coconut Shell Powder at 1.0kx, 2.0kx and 5.0kx amounting to 50 µm, 20 µm and 10 µm respectively. Fig. 5(D – F) show the micrographs of the samples after 20 minutes of milling at 1.0kx, 2.0kx and 5.0kx giving 50 µm, 20 µm and 10 µm respectively. Fig. 5(G – I) presented the micrographs of 40 min milling at 1.0kx, 2.0kx and 5.0kx producing 50 µm, 20 µm and 10 µm respectively while Fig. 5(J – L) represent the micrographs of 60 min milling at 1.0kx, 2.0kx and 5.0kx amounting to 50 µm, 20 µm and 10 µm respectively. The micrographs revealed that unmilled has the largest particle sizes when compare to other milled powder. The duration of milling greatly affects the distribution of the particles and how the crystalline are arranged.

It was revealed by EDX analysis, that the main constituents of CS-NPs are Carbon, Oxygen, Potassium, Calcium, Phosphorous, Copper, silicon and Chlorine as evidenced in Fig. 6. It has also been revealed by XRF that the main chemical composition constituents in coconut shell powder are CaO, Al_2_O_3_, MgO, K_2_O, Na_2_O, MnO, ZnO, SiO_2_ as well as Fe_2_O_3_.

Table 4 showed the main elemental constituents of CS-NPs and their variable percentage compositions. Carbon which is the major element has a range of 65.0 to 66.2%, Oxygen has percentage composition between 25.4 and 32.4%, Potassium is between 1.2 and 3.9%, and Chlorine is between 0.7 and 1.5%, Calcium composition ranges from 0.1 to 0.6%. While Silicon, Iron, Sodium, Sulphur, Magnesium are not detected at all milling phases and as such may not be empirically regarded as elements detected.

### 2.7 Data areas of application

i. Carbonaceous coconut shell nanoparticle can be used as reinforcement when carbonized during friction stir processing and welding [4] and can also find application in other reinforcements.
ii. It can be used as raw material for activated carbon industries. In all activated carbon producer, coconut shell has been proven to produce top grade active carbon charcoal which got great performance [5], [6], [7], [8].
iii. It can be used as filler for synthetic resin glues (thermoset moulding powder) in plywood and laminating board companies [9], [10].
iv. It can also be used as compound filler in the production of phenol formaldehyde moulding powder (Bakelite plastic) as filler and extender [11].
v. In South Asian countries, CS-NPs has been used as mosquito repellent by making it into mosquito coils as a burning medium and as a means of controlling fungus in the wood [12].
vi. It can be used as lost circulation materials in the oil well drilling companies [13].
vii. It can also be used in resin casting, mastic adhesives as well as bituminous products [14].
